# Learning-Based Non-Intrusive Electric Load Monitoring for Smart Energy Management

**DOI:** 10.3390/s24103109

**Published:** 2024-05-14

**Authors:** Nian He, Dengfeng Liu, Zhichen Zhang, Zhiquan Lin, Tiesong Zhao, Yiwen Xu

**Affiliations:** 1 Zhicheng College, Fuzhou University, Fuzhou 350002, China; 02121003@fdzcxy.edu.cn; 2 Fujian Key Lab for Intelligent Processing and Wireless Transmission of Media Information, Fuzhou University, Fuzhou 350108, China; 221120036@fzu.edu.cn (D.L.); 211120117@fzu.edu.cn (Z.Z.); n191127023@fzu.edu.cn (Z.L.); t.zhao@fzu.edu.cn (T.Z.)

**Keywords:** smart city, smart electric energy management, electric load monitoring, load recognition algorithm, computer vision

## Abstract

State-of-the-art smart cities have been calling for economic but efficient energy management over a large-scale network, especially for the electric power system. It is a critical issue to monitor, analyze, and control electric loads of all users in the system. In this study, a non-intrusive load monitoring method was designed for smart power management using computer vision techniques popular in artificial intelligence. First of all, one-dimensional current signals are mapped onto two-dimensional color feature images using signal transforms (including the wavelet transform and discrete Fourier transform) and Gramian Angular Field (GAF) methods. Second, a deep neural network with multi-scale feature extraction and attention mechanism is proposed to recognize all electrical loads from the color feature images. Third, a cloud-based approach was designed for the non-intrusive monitoring of all users, thereby saving energy costs during power system control. Experimental results on both public and private datasets demonstrate that the method achieves superior performances compared to its peers, and thus supports efficient energy management over a large-scale Internet of Things network.

## 1. Introduction

The past decades have witnessed a booming of urban populations with ever-increased municipal facilities to serve all citizens. An effective solution for managing these facilities is a smart city with an Internet of Things (IoT), which is mostly benefited from the recent development of Artificial Intelligence (AI) [1,2,3]. To support the smart city, an economic but efficient electric power management system is indispensable [4].

A cloud-end administrator monitors the electricity consumption of all users and loads, presents analyses of all electricity usages, and provides advice to users, or directly manages electricity usage of all loads. As a result, the overall electricity consumption rates are saved to support the sustainable development of cities and environments.

**Figure 1:**
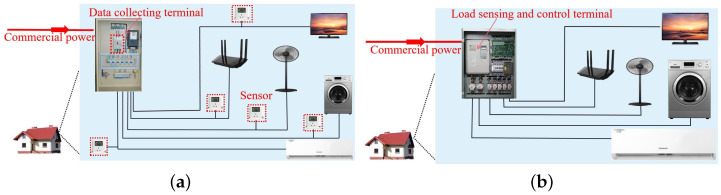
Intrusive and non-intrusive load monitoring in electricity grid. (**a**) Intrusive load monitoring. (**b**) Non-intrusive load monitoring.

An efficient electric power management system is dependent on its electric load monitoring module [5,6,7], which can be realized using intrusive or non-intrusive approaches. In intrusive load monitoring (ILM) [8,9], as shown in Figure 1a, each electric load is monitored by a separate sensor, and the information acquired from all sensors can be centrally processed at the cloud end. And in non-intrusive load monitoring (NILM) [6,7,10], as shown in Figure 1b, only one monitor is required for each family or cell. It captures electric signals (such as voltage, current, and so on) at the commercial power input and transmits them to the cloud server in which the workload information of all loads is interpreted with algorithms. Apparently, NILM is preferable in smart city infrastructure for its simple design, energy efficiency, and low setup/maintenance cost.

In the NILM design of Figure 1b, only one terminal is deployed at the access point of the family/cell. It observes the electrical loads in the room within a black box. How to design an effective load recognition algorithm (LRA) model to recognize or interpret these loads is thus critical.

Traditional LRA methodologies compared the feature of an unknown load with those of known loads in a dictionary. They made judgments through a metric set consisting of a matching degree [11], similarity degree [12], the Hellinger distance [13], etc. The performance of a LRA also benefited from the development of machine learning, resulting in recognition methods with K-means clustering [14] and fuzzy C-means clustering [15]. However, these methods basically utilized single features without considering subtle differences between similar signals. Therefore, the problem of recognition confusion has not been well addressed.

Researchers have considered introducing more types of signal features to improve the accuracy of the LRA. Ref. [16] proposed a load recognition model with a feature combination of a transient waveform and power change value during load switching. Kang et al. [17] employed a fast Fourier transform to extract the amplitude and phase of the odd harmonics of the current and then used them as key features for recognition. To improve the recognition accuracies of loads, Ref. [18] constructed a hybrid feature set using the parameters of active power, reactive power, and harmonic amplitude.

In the past decade, deep learning has demonstrated its strengths in AI-driven tasks, such as computer vision, natural language processing, human–computer interaction, and IoT. These successes have also inspired researchers to introduce deep neural networks to the LRA. Ref. [19] designed a sequence-to-sequence Long Short-Term Memory (LSTM) network for load recognition. The authors of [20] designed a capsule network-based LRA, in which a convolutional neural network (CNN) extracted latent features from a set of non-overlapping energy measurement data segments. Ref. [21] proposed a dual-stream neural network to extract features from current signals. Ref. [22] proposed to extract features with Siamese neural networks and then used them in load recognition. These works have revealed the strong feature extraction abilities of neural networks with promising performances in load recognition.

To further improve the LRA accuracy, researchers have also attempted to visualize the features of voltage or current and employed image-alike processing techniques in load recognition. Due to the advantage of the recent boom of computer vision technologies, more accurate and robust LRA methods have been developed. Ref. [23] presented an image classification-based LRA, where the image is obtained with voltage–current (V-I) trajectory. Ref. [24] provided a CNN-based LRA with weighted pixel V-I trajectory maps as features. Liu et al. employed a color-coded V-I trajectory map as the input of their AlexNet-based load recognition model [25]. In [26], the V-I trajectory and amplitudes of current and voltage were mapped as a color image, which provided richer feature information for CNN-based load recognition. Wenninger et al. [27] mapped a cycle of V-I trajectories as threshold-free recursive graphs and subsequently designed a Spatial Pyramid Pooling (SPP) convolutional neural network for load recognition.

**Figure 2:**
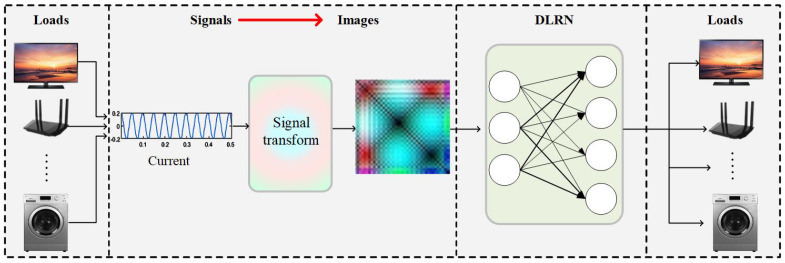
Non-intrusive load monitoring with feature visualization.

Despite these great efforts, there still exists a need to further improve the accuracy of the LRA. Generally, load recognition may lead to inaccurate results when using inappropriate feature extraction methods and neural networks or false recognizing loads under masking effect—features of low power load are usually hard to recognize under high power loads. To address these issues, an NILM method based on a more effective current feature visualization and a more reliable deep load recognition network (DLRN) is introduced in this study, as shown in Figure 2. The NILM method enables precise load recognition in smart homes, facilitating more effective energy management.

In summary, the main contributions are summarized as follows:

1. A method for the visualization of current features based on signal transformation and a Gramian Angular Field (GAF) is proposed. By this operation, the features difference between loads are highlighted to facilitate vision-based recognition.
2. This study presents a DLRN based on multi-scale feature extraction and an attention mechanism. This design aims to further enhance the recognition accuracy and generalization abilities of the NILM method, especially at low-power conditions.
3. The approach in this study demonstrates its high efficiency in both public and private datasets. To examine the generalization ability of the proposed approach, this study introduced a new dataset with 12 types of electric loads with powers from 24 W to 1800 W. The experimental results from using this dataset, as well as the public PLAID dataset, validate the design.

The rest of this paper is organized as follows. Section 2 presents a detailed discussion of the proposed learning-based NILM algorithm. Comprehensive experiments and analyses are presented in Section 3. Finally, Section 4 concludes this paper.

## 2. Proposed Method

### 2.1. Motivation and Framework

It is clear that the choice of input signal has a significant impact on the performance of the load recognition. Most traditional approaches [26,28,29] directly input signals such as voltage, current, and a V-I trajectory diagram into a feature extraction network and carry out the subsequent load recognition accordingly. However, studies [30,31,32] have shown that there is a large amount of noise with a great negative impact on current and voltage signals. This will lead to a decrease in the accuracy of subsequent recognition algorithm, which also leaves room for performance improvement. In other words, preprocessing the signal before inputting it into the recognition network is expected to improve the performance of the recognition algorithm.

Considering the excellent results of frequency domain analysis (e.g., wavelet transform, discrete Fourier transform) and signal processing, this study introduced them into the preprocessing of a novel method. Firstly, the current signal is decomposed into approximation and detail coefficients using a wavelet transform. The approximation coefficient represents the envelope information of the current signal, which can be used as the main information to distinguish the load; the detail coefficient represents the texture information of the current signal, which can distinguish the details of different loads and consequently improve the accuracy of the load recognition. Secondly, this study used the harmonic information of the coefficients for the load recognition to reduce the impact of noise on the recognition accuracy. The experimental results of this study show that harmonics are generated during the operation of power equipment, and the harmonics generated by different types of power equipment are different (see below for validation experiments).

**Figure 3:**
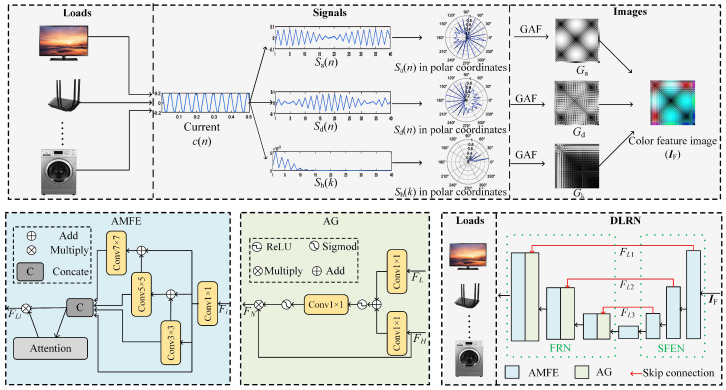
Flowchart of the proposed current feature visualization method and a DLRN.

Inspired by the above analysis, an NILM method is proposed, as shown in Figure 3. First of all, the method employs a wavelet transform and discrete Fourier transform (DFT) to decompose the current signal into three feature sequences: (i) an approximation coefficient sequence with an envelope feature of the current signal; (ii) a detail coefficient sequence with a texture feature of the current signal; and (iii) a harmonic ratio sequence with a harmonic feature of the current signal. After that, it utilizes the GAF method to convert the above three sequences into gray-scale images and further set them as the R, G, and B channels of a color image. Through this operation, the differences between loads are highlighted to facilitate load recognition. Finally, a DLRN based on multi-scale features and a visual attention mechanism is proposed. These steps will be elaborated on as follows.

### 2.2. Proposed Current Feature Visualization

In an NILM system, the terminal at the commercial power input is able to capture the voltage and current signals. Between them, the voltage remains almost intact while the current fluctuates with the electric usage on the loads. Therefore, the current signal is chosen as the input for the load recognition model. To visualize the current signal as a two-dimensional image, this study first extracted its features and then converted them into gray-scale images, which were further set as channels of the visualized color image.

Denote the current signal by c(n) with a length *N*. This can be expanded into a wavelet series as follows [33]:

(1) $$\begin{array}{c} c\left(n\right)=\frac{1}{\sqrt{N}}\sum_{k}W_{\varphi }\left(j_{0},k\right)\cdot \varphi_{j_{0},k}\left(n\right)+\\ \frac{1}{\sqrt{N}}\sum_{j=j_{0}}^{\infty }\sum_{k}W_{\psi }\left(j,k\right)\cdot \psi_{j,k}\left(n\right), \end{array}$$

where *j* represents the wavelet decomposition scale that determines the length of the wavelet coefficient, *k*. $\varphi_{j_{0},k}\left(n\right)$ and $\psi_{j,k}\left(n\right)$ represent the scaling and wavelet functions, respectively. $W_{\varphi }\left(j_{0},k\right)$ and $W_{\psi }\left(j,k\right)$ are the approximation coefficient and detail coefficient, respectively. For ease of presentation, this study denotes them as $S_{a}(n)$ and $S_{d}(n)$, n=0,1,2,…,N−1. They are calculated as follows [34]:

(2) $$\begin{array}{c} S_{a}\left(n\right)=\frac{1}{\sqrt{N}}\sum_{n}c\left(n\right)\cdot \varphi_{j_{0},k}\left(n\right). \end{array}$$

(3) $$\begin{array}{c} S_{d}\left(n\right)=\frac{1}{\sqrt{N}}\sum_{n}c\left(n\right)\cdot \psi_{j,k}\left(n\right),j>j_{0}. \end{array}$$

According to [35], $S_{a}\left(n\right)$ and $S_{d}\left(n\right)$ represent the envelope feature and texture feature of the current signal, respectively. This study used them as two features that will be visualized and recognized.

Another feature used in this paper is the harmonic content, which refers to the percentage of the *k*-th order harmonic component of the total harmonic components. As the *k*-th order harmonic component of the current signal can be obtained using a DFT [36],

(4) $$r(k)=\frac{1}{N}\sum_{n=1}^{N}c\left(n\right)\cdot e^{-j\frac{2\pi k}{N}n},$$

the harmonic content (denoted by $S_{h}\left(k\right)$) is formulated as follows:

(5) $$S_{h}\left(k\right)=\frac{r(k)}{\sum_{i=1}^{N}r\left(i\right)}.$$

**Figure 4:**
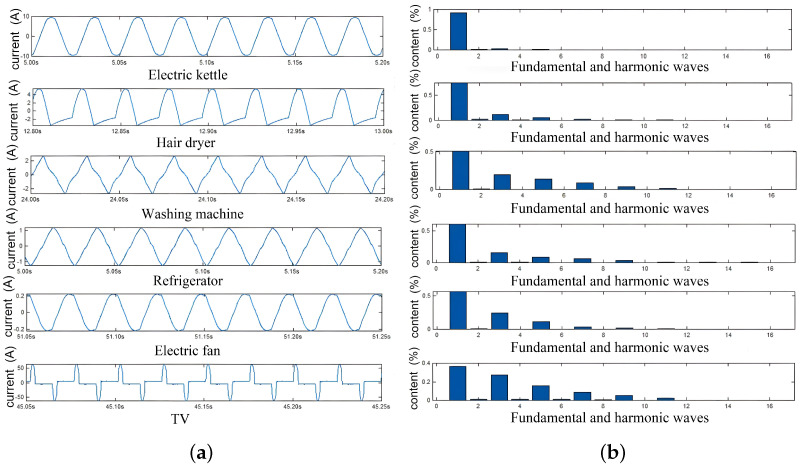
Current signals and harmonic contents of typical loads. (**a**) The current waves of typical loads. (**b**) The harmonic content of typical loads.

The harmonic contents of loads vary based on the electrical components and circuit systems. To verify the above assumption, this study explored the harmonic contents of different types of loads: resistive, pump-driven, motor-driven, and switching-powered. The results are shown in Figure 4, in which Figure 4a shows the current waves of typical loads, whereas Figure 4b shows their corresponding harmonic content. From this figure, the resistive loads (e.g., electric kettle, hair dryer) increase in heat with the resistors and barely have harmonic components. The pump-driven loads (e.g., washing machine, refrigerator) work mainly with a fundamental wave, but with more 3rd, 5th, and 7th harmonic components. The motor-driven loads (e.g., electric fan) are similar to pump-driven loads. They have 3rd, 5th, 7th, and 9th harmonic components that are lower the than fundamental wave. The switching-powered loads (e.g., TV, computer) adjust output voltages with high-frequency switches. They generate rich high-order harmonic components, such as 3rd, 5th, 7th, 9th, 11th, and 13th harmonic components, which are comparable with the fundamental wave. Obviously, the harmonic content, i.e., $S_{h}\left(k\right)$, is an effective feature used to recognize different types of loads.

Then, this study mapped all extracted features of $S_{a}\left(n\right)$, $S_{d}\left(n\right),$ and $S_{h}\left(k\right)$ into gray-scale images with the GAF method. Take $S_{a}\left(n\right)$ for example. Firstly, $S_{a}\left(n\right)$, where n=0,1,2,…,N−1, is transferred from the Cartesian coordinate system to a polar coordinate system:

(6) $$\left\{\begin{array}{c} \varphi \left(n\right)=\mathrm{arctg}\left[S_{a}\left(n\right)\right]\\ r\left(n\right)=\sqrt{{S_{a}}^{2}\left(n\right)+n^{2}} \end{array}\right..$$

Then, an n×n Gramian matrix $G_{a}$ is obtained in which

(7) $$G_{a}\left(i,j\right)=\sin\left(\varphi \left(i-1\right)-\varphi \left(j-1\right)\right).$$

Similarly, the Gramian matrixes of $S_{d}\left(n\right)$ and $S_{h}\left(k\right)$ are also obtained and are, respectively, denoted by $G_{d}$ and $G_{h}$. This study used the following equations to map these Gramian matrixes to the R, G, and B channels of a color image:

(8) $$\left\{\begin{array}{c} R=\left|G_{a}\right|\times 255\\ G=\left|G_{d}\right|\times 255\\ B=\left|G_{h}\right|\times 255 \end{array}\right.,$$

(9) $$I_{F}=\left[R\;G\;B\right].$$

**Figure 5:**
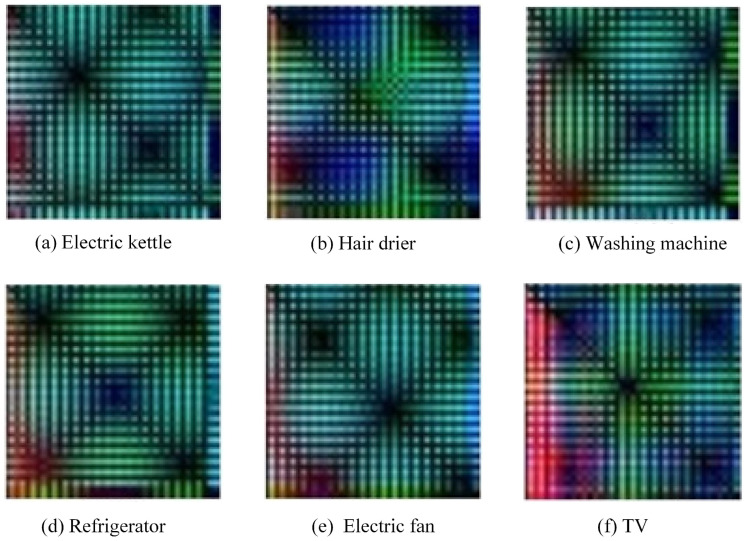
The results of current feature visualization for typical loads.

The flowchart of the current feature visualization proposed in this study is summarized at the top of Figure 3. The results of the current feature visualization for typical loads (including an electric kettle, hair drier, washing machine, refrigerator, electric fan, and TV) are shown in Figure 5. It can be seen from this figure that after feature visualization, the fused color image (i.e., $I_{F}$), with a resolution of 40 × 40, has its unique texture information and chroma components. It includes low-frequency envelope features, high-frequency texture details, and all harmonic ratios. Compared with the original one-dimensional current signal, it visualizes and highlights all hidden features whilst keeping the timestamp of the original signal. As a result, this study was allowed to use CNN methods to recognize all loads with a high accuracy.

### 2.3. Proposed DLRN

#### 2.3.1. Overview of the Network

To recognize a load from $I_{F}$, a critical issue is how to effectively extract both global and local features and avoid the influence of noise. To this end, a deep feature extraction neural network (i.e., DLRN) was designed in this study, which is illustrated at the bottom of Figure 3. The DLRN consists, primarily, of a Shallow Feature Extraction Network (SFEN), an intermediate layer module, a Feature Recombination Network (FRN), and skip connections. The SFEN employs continuous attention-based multi-scale feature-dense extraction modules (denoted by AMFEs) to extract various types of feature information from the $I_{F}$. The intermediate layer AMEF further processes the output of the SFEN to capture important load features. To preserve shallow features, the output of each AMEF (denoted as $F_{Li}$) is fed into the FRN through skip connections:

(10) $$F_{Li}=\left\{\begin{array}{cc} M(I_{F}) & i=1\\ M\left(F_{L\left(i-1\right)})\;\right. & i=2,3 \end{array}\right.,$$

where $F_{Li}$ represents the output of the *i*-th layer’s AMFE, and M() represents the AMFE. In the FRN, Attention Gates (AGs) and AMEFs make progress between shallow and crucial load features. Ultimately, the output from the FRN is subjected to average global pooling, a fully connected layer, and a softmax function for precise load recognition.

#### 2.3.2. AMFE

In order to effectively extract features about envelope information, texture details, and all harmonic ratios in load images, the AMFEs in both the SFEN and FRN were designed in this study, which are depicted in Figure 3. First, an AMFE utilizes a 1 × 1 convolution to extract coarse-grain features and further divides them into four feature sub-sets with the same space size. Second, it employs four different convolution kernels to extract features from different sub-sets:

(11) $$b_{i}=\left\{\begin{array}{cc} a_{i} & i=1\\ m_{i}\left(b_{i-1}+a_{i}\right) & 2\leq i\leq 4 \end{array}\right.,$$

where $m_{2}\left(\right)$, $m_{3}\left(\right)$, and $m_{4}\left(\right)$ are 3 × 3, 5 × 5, and 7 × 7 convolutions, respectively. Third, it concatenates all inputs $b_{i}$, i∈[1,4] and uses a 1 × 1 convolution to fuse all features from different scales. Finally, it employs an attention mechanism to adaptively allocate weights to multi-scale features. The attention mechanism allows the network to adaptively adjust the importance of each channel. By weighting operations on the feature maps, the attention mechanism highlights important features and suppresses the other features; thus, it degrades the impacts of non-relevant features in load recognition.

In using AMFE, the proposed network deeply exploits the image features at different scales that benefit the feature extraction ability of the model.

#### 2.3.3. Attention Gate

During the process of the FRN, low-level load features will be replaced by high-level load features. To address this issue, an AG is introduced before each addition operation to better recombine high-level and low-level features. Figure 3 shows the network structure of an AG, which incorporates both low-level and high-level features for load recognition. In this structure, the low-level feature from a skip connection ($F_{L}$) serves as a gate control signal, while a high-level feature ($F_{H}$) acts as an input signal. To combine these two signals, both signals are separately convoluted 1 × 1 and added into a new signal. Then, the new signal is activated by the ReLU function, convoluted 1 × 1, and filtered by a Sigmoid function to obtain a weight matrix. Finally, the feature $F_{H}$ is multiplied element-wise with the weight matrix obtained earlier, resulting in a new feature $F_{N}$ that integrates both low-level and high-level features for load recognition. The features are passed through the AG network, which can be represented by the following:

(12) $$\alpha_{t}=\varphi_{1}\left(f^{1\times 1}\left(\varphi_{2}\left(f^{1\times 1}\left(F_{L}\right)+f^{1\times 1}\left(F_{H}\right)\right)\right)\right),$$

(13) $$F_{N}=F_{H}*\alpha_{t},$$

where $f^{1\times 1}\left(\right)$ denotes a convolution of 1 × 1, $\varphi_{1}$ represents a Sigmoid function, $\varphi_{2}$ represents the ReLU function, and $\alpha_{t}$ is the weight matrix of the AG network.

In incorporating the AG network, the model selectively enhances important low-level features while preserving the information from high-level features.

### 2.4. The Overall Algorithm for the Proposed NILM

In summary, the steps of the proposed NILM method are as follows:

Step 1. Obtain the current signal c(n).

Step 2. Calculate the three feature sequences, approximation coefficient $S_{a}\left(n\right)$, detail coefficient $S_{d}\left(n\right),$ and harmonic content $S_{h}\left(k\right)$ with Equations (2), (3), and (5).

Step 3. Use the GAF method, as shown in Equations (6)–(9) to convert these feature sequences into a color image $I_{F}$.

Step 4. Recognize the electric load with the image $I_{F}$ and the proposed DLRN in Figure 3.

## 3. Experiments and Simulations

To examine the performance of the method, this study compared it with state-of-the-art methods on popular datasets. Ablation studies were also conducted in this study to validate the effectiveness of the network design. In addition, this study discusses the application of the proposed method of NILM in smart cities.

### 3.1. Datasets

This study compared all methods in a publicly available dataset, Plug Load Appliance Identification Dataset (PLAID) [37], with electric usage data of 11 types of loads. To increase the data samples for training, this study also designed a load-sensing terminal and captured the electric usage of 12 types of popular loads. The powers of these loads ranged from 24 W to 1800 W, which are representative for testing the LRAs under similar electric loads or the masking effect of other loads.

### 3.2. The Performance of the Method in This Study

The results of the proposed method are summarized in Table 1, including results on both the PLAID and the private dataset. For each dataset, this study split it based on a 70:30 ratio for training and testing. From the table, the method achieved a promisingly high accuracy of 98.26% on the private dataset with an F1 score of 0.9819. It also achieved an accuracy of 0.9771 and an F1 score of 0.9743 on the PLAID. These results fully demonstrate the efficiency of the load recognition model in this study.

**Figure 6:**
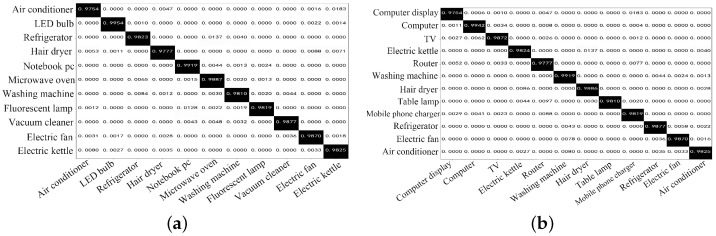
Confusion matrixes of the method. (**a**) Results on the private dataset. (**b**) Results on the PLAID.

To analyze the recognition accuracies of different types of loads, confusion matrixes of testing sets are displayed in Figure 6. In Figure 6, all resistive loads, e.g., electric kettle (1800 W) and hair dryer (1200 W), can be well recognized with a high accuracy of 98.20%. This fact is mainly attributed to their distinct features of high powers and low harmonic content. As discussed in Section 2, pump-driven loads have similar feature distributions to those of motor-driven loads. In these categories, the model proposed in this study successively recognized the washing machine, electric fan, air conditioner, and vacuum cleaner with a low probability of an incorrect recognition or confusion. Switching-powered loads have high harmonic content, but they also have low powers that might be covered by high-power loads. Based on Figure 6, the method in this study is well capable of addressing this issue. Its recognition accuracies of the notebook, TV, and router exceed 96.88% even when high-power loads (e.g., electric kettle, air conditioner) are working at full powers.

### 3.3. Comparison with Popular Methods

To examine the superiority of the proposed method, this study compared it with those of references [24,25,26] under the same conditions. The evaluation results on the PLAID are shown in Table 2. From the table, the proposed method surpasses all compared algorithms on the PLAID with an average accuracy of 97.71%. This fact validates the effectiveness of the proposed method in electric load recognition. It is thus capable of identifying all types of loads after fine training in a large-scale electricity management system for a smart city.

In addition, both two stages of the model in this study, current feature visualization and deep load recognition, contribute to the final performance. With the current feature visualization and Ding’s CNN model, the hybrid method also achieves an accuracy of 96.91% that outperforms Ding’s method. By using both current feature visualization and deep load recognition, the method in this study outperforms Ding’s by 1.08%. This fact also validates the design.

### 3.4. Ablation Study

Ablation experiments were also performed in this study to examine the design of the DLRN. Experiments were run on both the PLAID and private dataset.

*(1) The Effectiveness of the Feature Extraction*: In this method, this study employed an approximation coefficient, detail coefficient, and harmonic ratio as the three features for load recognition, as shown at the top of Figure 3. To validate the effectiveness of these features, this study implemented an experiment in which the features used in the traditional methods [26,28,29] were fed to the proposed network, and then the traditional method’s performances was compared with that of the proposed method. The results are shown in Table 3. From this table, it can be seen that the proposed feature extraction method achieves an apparently higher accuracy compared to the traditional method.

*(2) The Effectiveness of Multi-Scale Feature Extraction*: The proposed deep network utilizes multi-scale convolution kernels (1 × 1, 3 × 3, 5 × 5, 7 × 7), as shown in the network part of Figure 3. To validate their effectiveness, this study compared them with identical kernel settings, e.g., 1 × 1, 3 × 3, 5 × 5, or 7 × 7, and the results are summarized in Table 4. All settings were retrained for a fair comparison. From the table, the multi-scale feature extraction design is superior to all other settings. Therefore, the proposed deep model can well extract all critical information from the visualized current and, thus, is more suitable for load recognition.

*(3) The Effectiveness of AGs*: The AGs are utilized to add multi-scale features after skip connections. From Table 5, the proposed method achieves an inferior performance without these AGs. Therefore, both multi-scale feature extraction and the attention mechanism contribute to the final recognition performance.

### 3.5. Discussion on Practical Use

**Figure 7:**
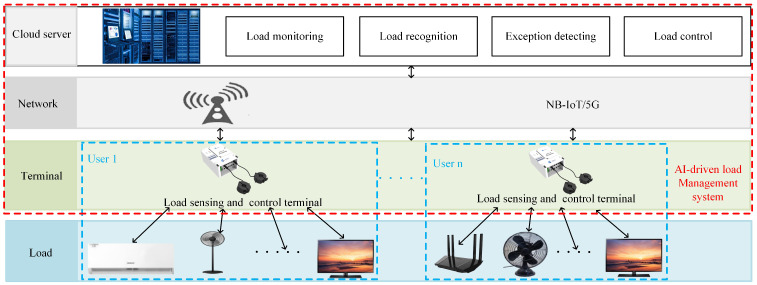
An application paradigm of the method in this study.

A practical AI-driven load management system can be implemented with the proposed load recognition method, as shown in Figure 7. This system was designed as a joint Terminal–Network–Cloud infrastructure for smart cities.

At the terminal end, a load-sensing and control terminal is in charge of managing all loads in a family or cell. It collects load information (e.g., the current signal) and sends it to a cloud server for further analysis. It also receives and executes all commands from the cloud server to control loads for energy saving. The network is utilized to transmit all data and commands, which can be based on technologies like Narrow Band IoT (NB-IoT) or 5G.

At the cloud end, a cloud-based service platform is responsible for load recognition, monitoring, and control. It analyzes the electricity consumption of all loads and provides suggestions for the smart control of switches. In controlling the loads, unnecessary (e.g., lighting under daylight) or dangerous (e.g., abnormal use in factory) uses of electricity can be avoided. The cloud-based service platform runs the proposed NILM method only when necessary, so the computational cost is not a critical issue. The experiments also demonstrate that the NILM approach can run on a laptop, as the proposed deep model processes a 40 × 40 color image only.

A laboratory prototype was designed to assert the above application paradigm. It was a minimum system consisting of the load-sensing and control terminal and the application software. When the system starts working, it firstly checks the connection status of the load sensing and control terminal. The information (such as serial number, IP address, etc.) of the online terminals is displayed in the application software. Meanwhile, the current data, which are captured and transferred by the online terminals, are received and stored in the server by the application software. Then, the application software uses the proposed NILM algorithm for load recognition. The results are employed to determine whether there are abnormal loads (e.g., unnecessary loads) based on a predefined mechanism. When an abnormality occurs, the abnormality information is sent to the terminal, which subsequently performs a corresponding operation (e.g., turn off the unnecessary load). It is worth mentioning that the above laboratory prototype was also utilized to establish the private dataset mentioned in Section 3.1.

In summary, the joint Terminal–Network–Cloud infrastructure enables efficient electricity management over a large-scale IoT in smart cities, allowing for load monitoring, analysis, and control for each family or cell. The proposed NILM approach can be deployed in the smart electricity management system of smart cities.

## 4. Conclusions

This paper proposes an NILM method based on a current feature visualization and deep recognition network. It first converts current signals into color images with a combination of a signal transform and thhe GAF method. Then, it utilizes multi-scale feature extraction and attention mechanisms to form a deep recognition network. The experimental results demonstrate that the proposed algorithm surpasses all compared algorithms on the public PLAID with an average accuracy of 97.71%. The proposed method can be embedded into a load monitoring and management system to control the electricity usage and save energy in a smart city. It is worth noting that load recognition and management pose certain challenges in practical application. For example, there is no specific legal framework supporting the use of a load recognition system in different application scenarios (e.g., private homes, enterprises, public organizations). Additionally, the legal mechanisms for managing electronic receivers remain unclear. This might involve whether the terminal provides users with email recommendations or directly shuts down loads. These are proposed as future works.

## Figures and Tables

**Table 1:** Performance of the method.

Database	Accuracy	Precision	Recall	F1
Private	0.9826	0.9818	0.9821	0.9819
PLAID	0.9771	0.9779	0.9707	0.9743

**Table 2:** Comparison results on the PLAID.

Method	Network	Accuracy (%)
De’s [24]	CNN	91.74
Liu’s [25]	AlexNet	95.40
Ding’s [26]	CNN	96.63
Proposed	DLRN	97.71

**Table 3:** Effectiveness of the features employed in the proposed method.

	Accuracy	Precision	Recall	F1
Traditional	0.9273	0.9282	0.9273	0.9259
Proposed	0.9826	0.9818	0.9821	0.9819

**Table 4:** Performance evaluation with different kernels.

Kernel	Accuracy	Precision	Recall	F1
1 × 1	0.85100	0.85611	0.85076	0.85342
3 × 3	0.92458	0.92573	0.92184	0.92378
5 × 5	0.95333	0.95576	0.94941	0.95257
7 × 7	0.95497	0.95720	0.95393	0.95566
Proposed	0.98465	0.98681	0.98114	0.98396

**Table 5:** Performance evaluation w/ and w/o AGs.

	Parameters	Accuracy	Precision	Recall	F1
w/ AG	1264014	0.98465	0.98681	0.98114	0.98396
w/o AG	1247515	0.97497	0.97481	0.97235	0.97357

## Data Availability

Some or all data, models, or code generated or used during the study are available from the corresponding author upon request.
